# In vivo distribution of cerebrospinal fluid tracer in human upper spinal cord and brain stem

**DOI:** 10.1172/jci.insight.173276

**Published:** 2023-12-08

**Authors:** Erik Melin, Are Hugo Pripp, Per Kristian Eide, Geir Ringstad

**Affiliations:** 1 Department of Radiology, Østfold Hospital Trust, Grålum, Norway.; 2 Institute of Clinical Medicine, Faculty of Medicine, University of Oslo, Oslo, Norway.; 3 Oslo Centre of Biostatistics and Epidemiology, Research Support Services, Oslo, Norway.; 4 Faculty of Health Sciences, OsloMet - Oslo Metropolitan University, Oslo, Norway.; 5 Department of Neurosurgery and; 6 Department of Radiology, Oslo University Hospital-Rikshospitalet, Oslo, Norway.; 7 Department of Geriatrics and Internal medicine, Sorlandet Hospital, Arendal, Norway.

**Keywords:** Neuroscience, Drug therapy, Neurological disorders

## Abstract

**BACKGROUND:**

Intrathecal injection is an attractive route through which drugs can be administered and directed to the spinal cord, restricted by the blood-spinal cord barrier. However, in vivo data on the distribution of cerebrospinal fluid (CSF) substances in the human spinal cord are lacking. We conducted this study to assess the enrichment of a CSF tracer in the upper cervical spinal cord and the brain stem.

**METHODS:**

After lumbar intrathecal injection of a magnetic resonance imaging (MRI) contrast agent, gadobutrol, repeated blood samples and MRI of the upper cervical spinal cord, brain stem, and adjacent subarachnoid spaces (SAS) were obtained through 48 hours. The MRI scans were then analyzed for tracer distribution in the different regions and correlated to age, disease, and amounts of tracer in the blood to determine CSF-to-blood clearance.

**RESULTS:**

The study included 26 reference individuals and 35 patients with the dementia subtype idiopathic normal pressure hydrocephalus (iNPH). The tracer enriched all analyzed regions. Moreover, tracer enrichment in parenchyma was associated with tracer enrichment in the adjacent SAS and with CSF-to-blood clearance. Clearance from the CSF was delayed in patients with iNPH compared with younger reference patients.

**CONCLUSION:**

A CSF tracer substance administered to the lumbar thecal sac can access the parenchyma of the upper cervical spinal cord and brain stem. Since CSF-to-blood clearance is highly individual and is associated with tracer level in CSF, clearance assessment may be used to tailor intrathecal treatment regimes.

**FUNDING:**

South-Eastern Norway Regional Health and Østfold Hospital Trust supported the research and publication of this work.

## Graphical abstract

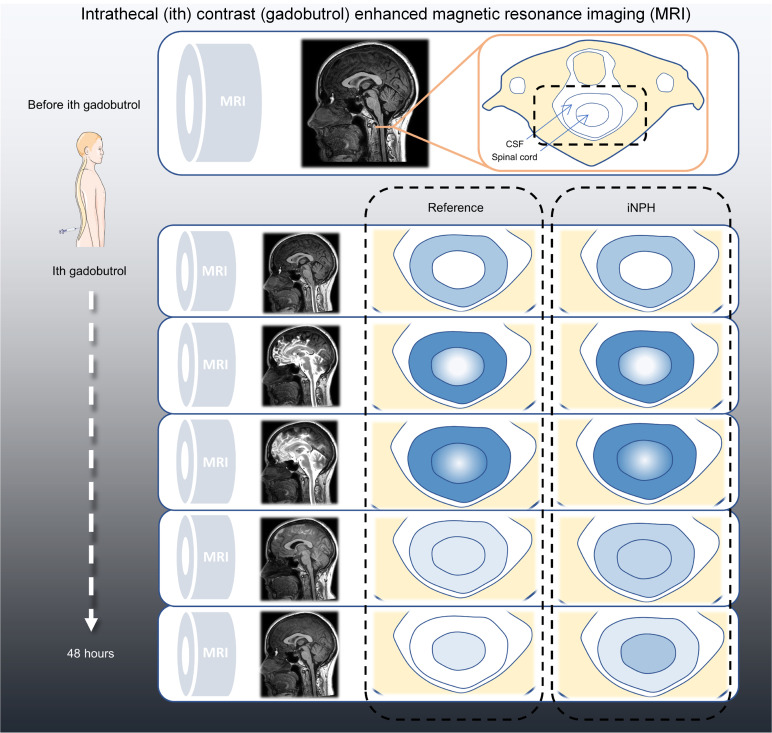

## Introduction

Drug delivery to the CNS is limited by the blood-brain barrier (BBB). Different technologies to bypass the BBB include disrupting this barrier, transnasal drug delivery, and intrathecal drug delivery to the cerebrospinal fluid (CSF) (1). Intrathecal drug delivery to the CNS has a long tradition for treatment of pain and spasticity (2, 3) and is increasingly being investigated for treatment of different neurodegenerative diseases targeted both on the brain and on the spinal cord — for example, Huntington′s disease, spinal muscular atrophy (SMA), and amyotrophic lateral sclerosis (4–7).

We know from studies of the brain that a small substance in the CSF can enrich all regions of the human brain (8). The inflow of CSF to the brain parenchyma is possibly a mix of diffusion and convection along perivascular spaces (9) facilitated by arterial pulsations (10). A similar system with perivascular influx of CSF from the subarachnoid space (SAS) to the rodent spinal cord exists (11–14). However, human data on the exchange between the CSF and the spinal cord are limited to a few autopsy cases (15), and pharmacokinetic data after intrathecal injections are incomplete (Table 1). A better understanding of the distribution of CSF substances and CSF physiology is needed for the assessment of new drugs and dosage in different patient groups according to age and disease and could have implications for diagnosing or treating conditions related to CSF, including tumors, degenerative or inflammatory diseases, and syringomyelia.

Patients with the CSF circulation disorder idiopathic normal pressure hydrocephalus (iNPH), a subtype of dementia, show delayed clearance of a CSF tracer from the brain compared with younger reference patients (8, 16). The mechanism behind this observation is incompletely understood, but reduced vessel wall pulsatility and loss of aquaporin 4 water channels with increasing age are associated with impairment of the perivascular clearance (17).

Therefore, we designed this observational magnetic resonance imaging (MRI) study to assess to which extent a CSF tracer enriches the human upper spinal cord and brain stem in vivo over 48 hours. Furthermore, tracer enrichment in patients with iNPH was compared with a reference group of patients with no verified CSF circulation disorder.

## Results

### Patients.

After the evaluation at the neurosurgery department, patients with shunt-responsive iNPH according to Japanese guidelines were placed in the iNPH category (*n* = 35), and patients without any evidence of CSF disorder were placed in the reference category (*n* = 26). The patient demographics are presented in Table 2. There was a higher proportion of females and a lower average age in the reference group compared with the iNPH group.

All patients have been included in earlier studies of CSF tracer distribution and CSF clearance from our group. The CSF tracer data for the enrichment in brainstem and the spinal cord have never been published before.

### Distribution of CSF tracer in reference patients.

**Figure 1:**
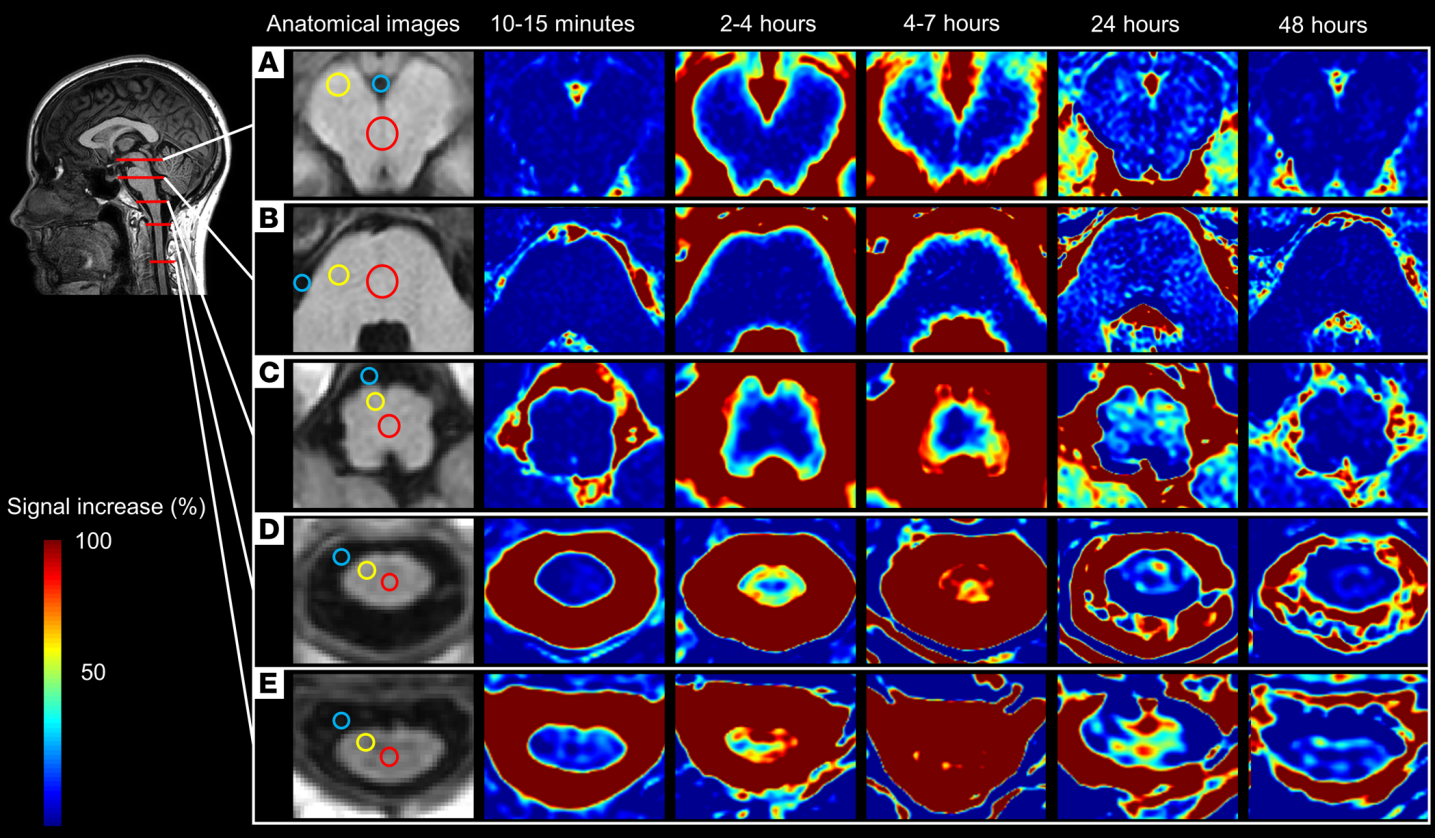
Tracer distribution over time and regions of interest for one reference patient. (**A**–**E**) Images of all time points and anatomical regions: mesencephalon (**A**), pons (**B**), medulla oblongata (**C**), C1 level of the spinal cord (**D**), and C3-level of the spinal cord (**E**). The 3D T1 images for all time points were first registered using Philips IntelliSpace, version 12.1. The signal in the T1-weighted images before contrast was then subtracted from the normalized signal for each time point. The tracer moves in a centripetal direction with higher and earlier detected levels of tracer in the superficial regions compared with the deep regions. For each time point, 15 regions of interest (ROIs) were placed on axial T1-weighted images in the hospital’s picture and archiving system (Sectra, version 7). The ROIs for each region are marked with circles in the anatomical images: blue represents ROIs in cerebrospinal fluid, yellow represents ROIs in the superficial parts of the parenchyma, and red represents ROIs in the deep parts of the parenchyma.

**Figure 2:**
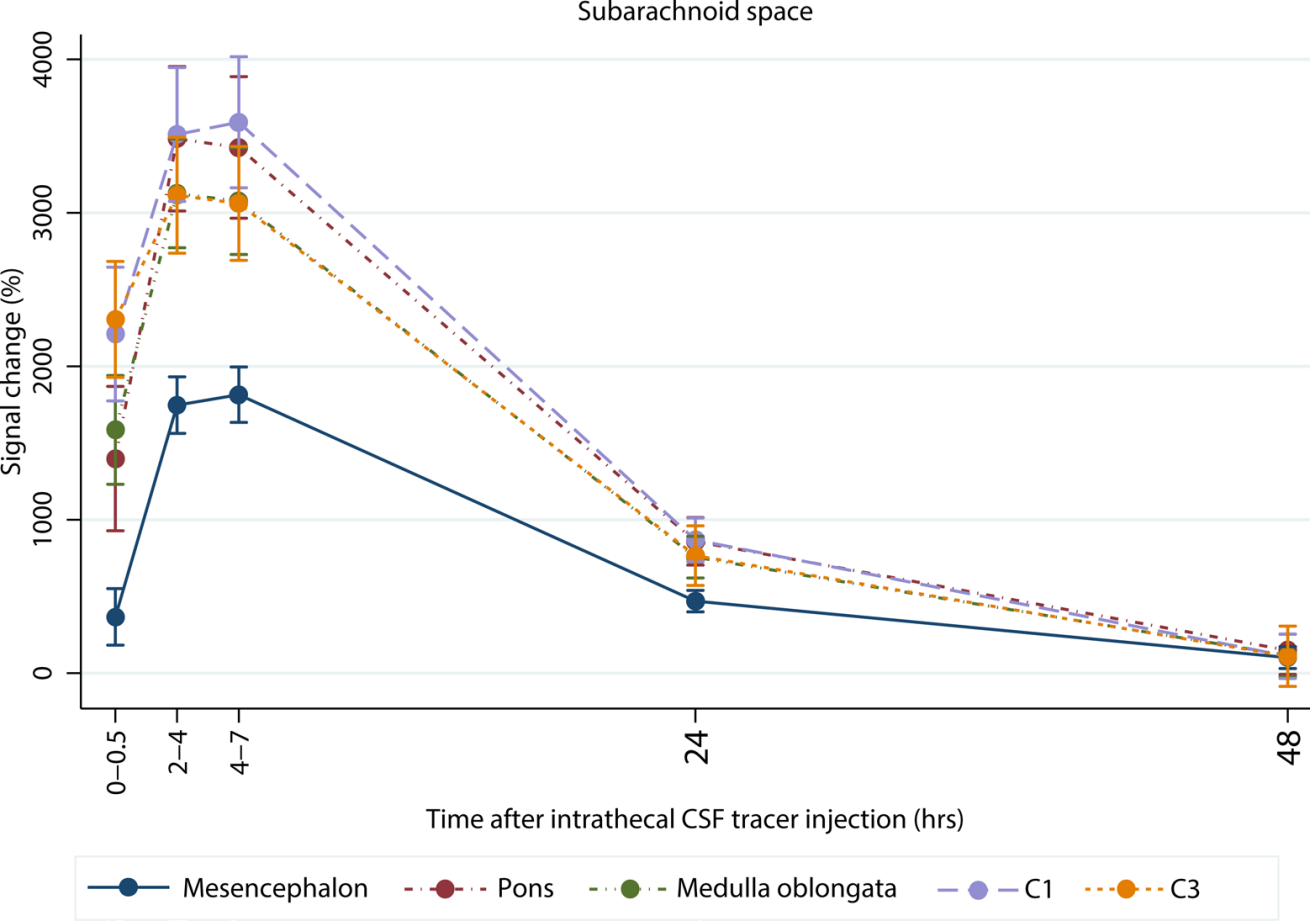
Tracer enrichment in the subarachnoid space for reference patients. Percentage change in normalized T1-signal over time after intrathecal CSF tracer injection for the subarachnoid space at the level of the mesencephalon, pons, medulla oblongata, and the C1 and C3 levels at the spinal cord for reference patients (*n* = 26). The data are presented as mean with error bars showing 95% CI (mixed-model analysis).

**Figure 3:**
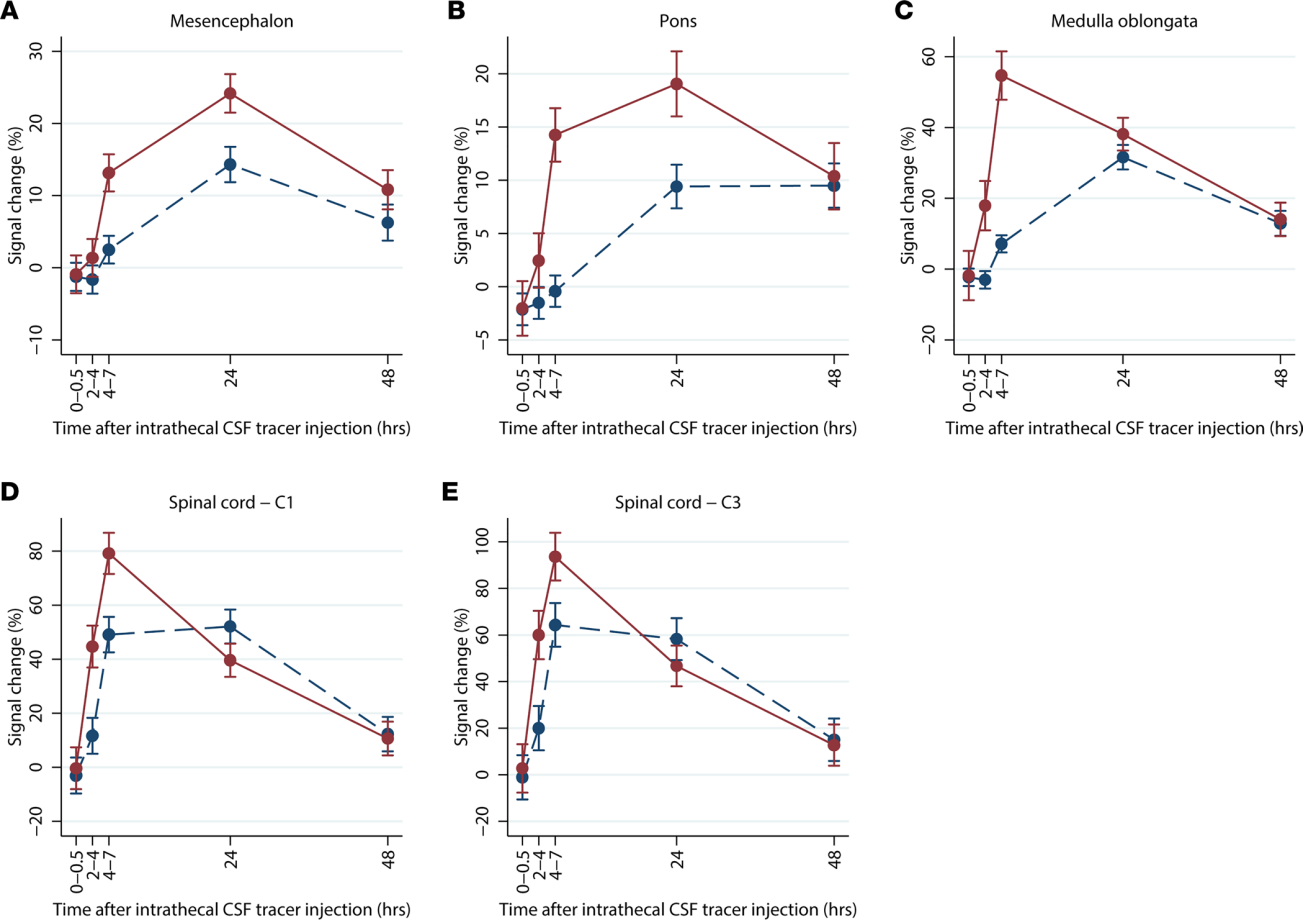
Tracer enrichment in deep and superficial parts for reference patients. (**A**–**E**)Percentage change in normalized T1-signal over time for mesencephalon, pons, medulla oblongata, C1 level of the spinal cord, and C3-level of the spinal cord for reference patients (*n* = 26). Red lines represent the superficial regions, and blue dotted lines represent the deep regions. The data presented as mean with error bars showing 95% CI (mixed-model analysis).

**Figure 4:**
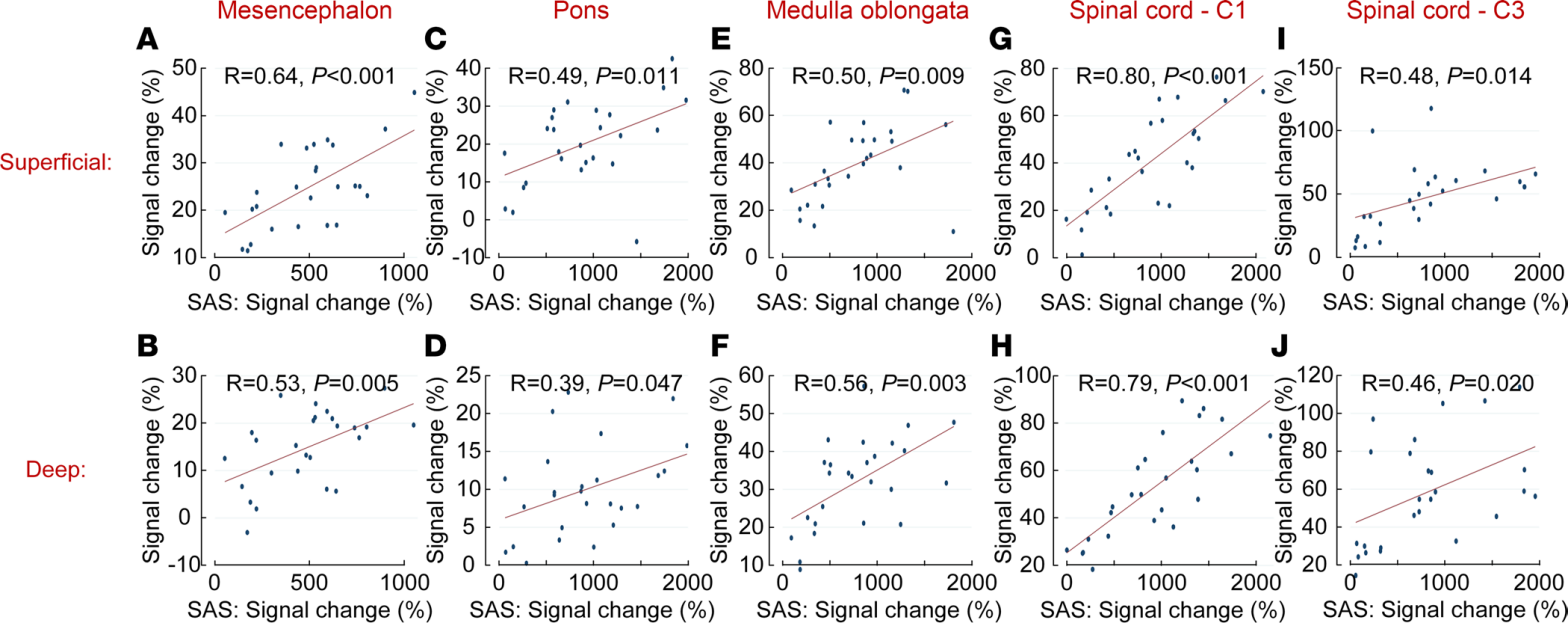
Correlations between CSF tracer in the subarachnoid space (SAS) and the superficial and deep regions of the parenchyma in reference patients (*n* = 25) after 24 hours. (**A**–**J**) Pearson correlation coefficients and significance levels are given. All regions show a significant correlation (*P* < 0.05). Pearson’s correlation coefficient was used.

Figure 1 shows the distribution of the CSF tracer in SAS, brainstem, and upper cervical spinal cord over time for 1 reference patient. In the first 4–7 hours, the tracer reached all levels of the SAS in approximately the same amounts, except for the most cranial part, anterior to the mesencephalon, where less tracer was detected (Figure 2). After 48 hours, most of the CSF tracer had been cleared from the SAS, except for anterior to the mesencephalon, where low levels of tracer were still detectable (102% signal increase, *P* = 0.005). Tracer enrichment was found in all parenchymal regions (Figure 3) and was higher in superficial parts compared with deep regions and higher in the spinal cord compared with the brainstem. After 48 hours, there were remains of tracer in the parenchyma. The tracer enrichment in the parenchyma was highly associated with tracer enrichment in the adjacent SAS after 24 hours (Figure 4).

### CSF-to-blood tracer clearance in reference patients.

Correlations between different pharmacokinetic variables of CSF-to-blood and tracer enrichment in SAS and parenchyma after 24 hours are presented in Table 3. The pharmacokinetic variable with the strongest association with CSF enrichment was the maximum concentration (C_max_) of tracer in blood, but also the time to 50% tracer dose absorbed to blood (T_1/2, abs_) and time to maximum concentration (T_max_) of tracer in blood showed strong associations with tracer enrichment in the SAS.

### Distribution of CSF tracer in patients with iNPH dementia.

**Figure 5:**
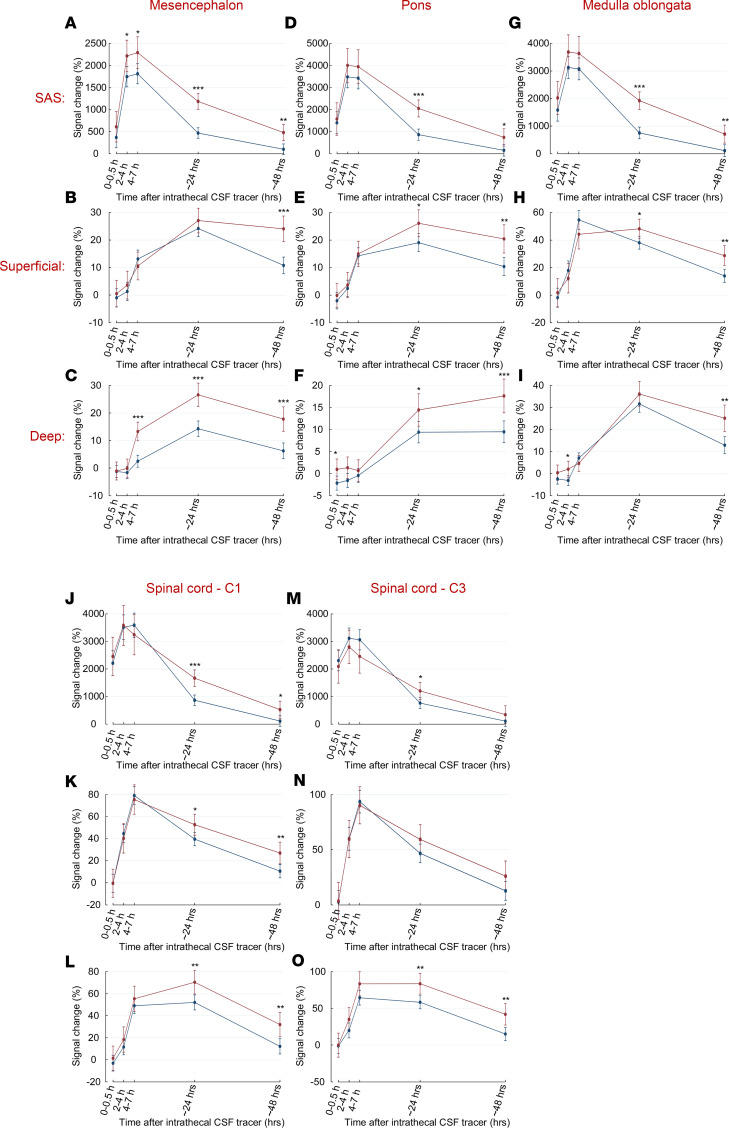
Tracer enrichment in patients with idiopathic normal pressure hydrocephalus (iNPH) and reference patients over time. (**A**–**O**) For several locations, patients with iNPH (red lines) (*n* = 35) had stronger enrichment in both the subarachnoid space (SAS) and the parenchyma compared with reference patients (blue lines) (*n* = 26). Data are shown as mean with error bars showing 95% CI. *P < 0.05, **P < 0.01, ***P < 0.001 (mixed-model analysis).

For several locations in both SAS and the parenchyma, the tracer enrichment was stronger in the iNPH group compared with the reference group (Figure 5). In SAS, the difference in tracer enrichment was largest after 24 hours. In the parenchyma, the difference was largest after 48 hours.

### Age and CSF tracer enrichment.

**Figure 6:**
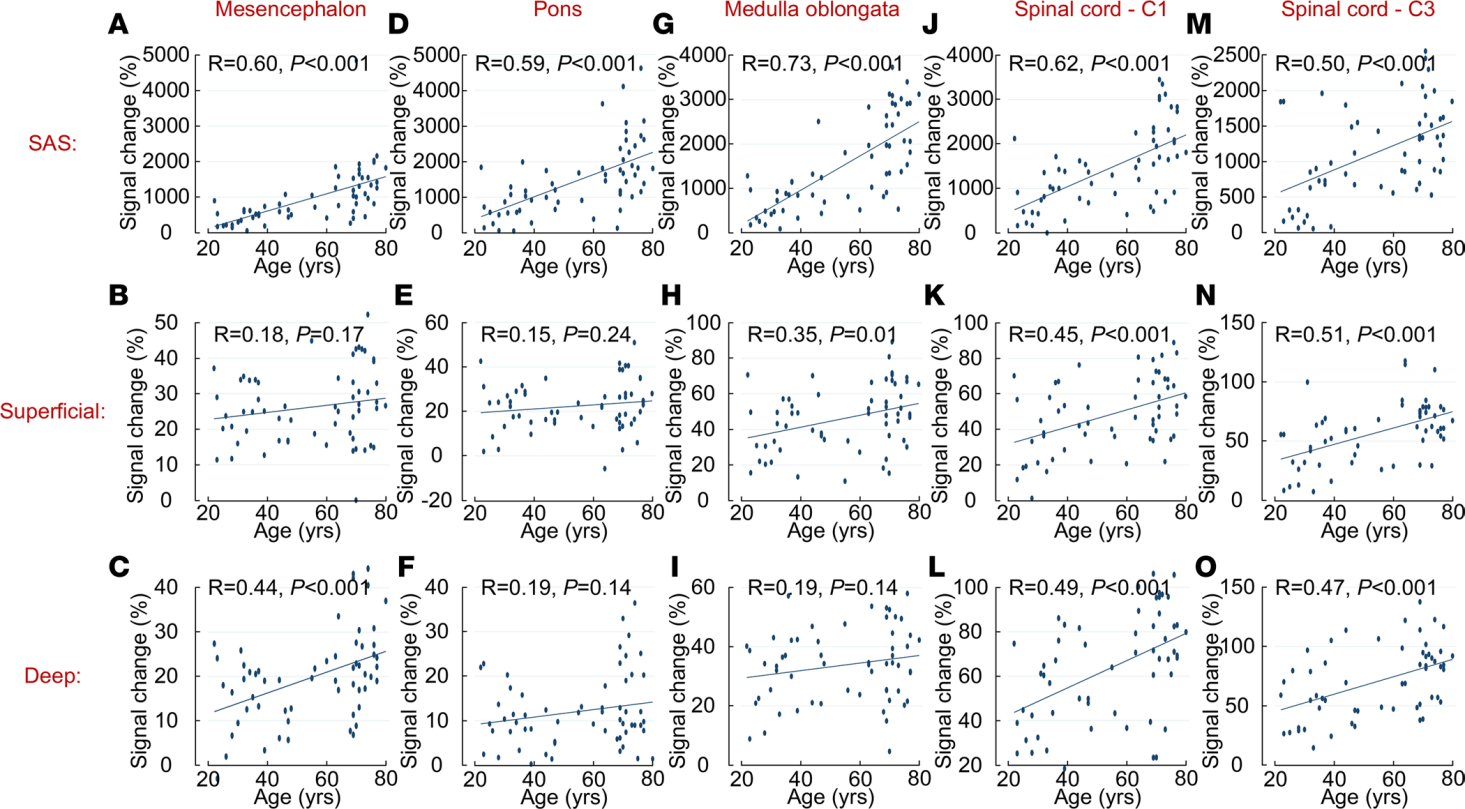
(A–O) Correlation between age and tracer enrichment after 24 hours for the subarachnoid space (SAS) and superficial and deep regions of the parenchyma. Both reference patients (*n* = 26) and patients with idiopathic normal pressure hydrocephalus (*n* = 31) are included. Pearson correlation coefficients and significance levels are given. Pearson correlation coefficient was used.

For all patients, there was a strong correlation between age and SAS enrichment after 24 hours (Figure 6). The correlations between age and parenchymal enrichment were highly significant for the cervical spinal cord.

### CSF-to-blood clearance and CSF tracer enrichment.

**Figure 7:**
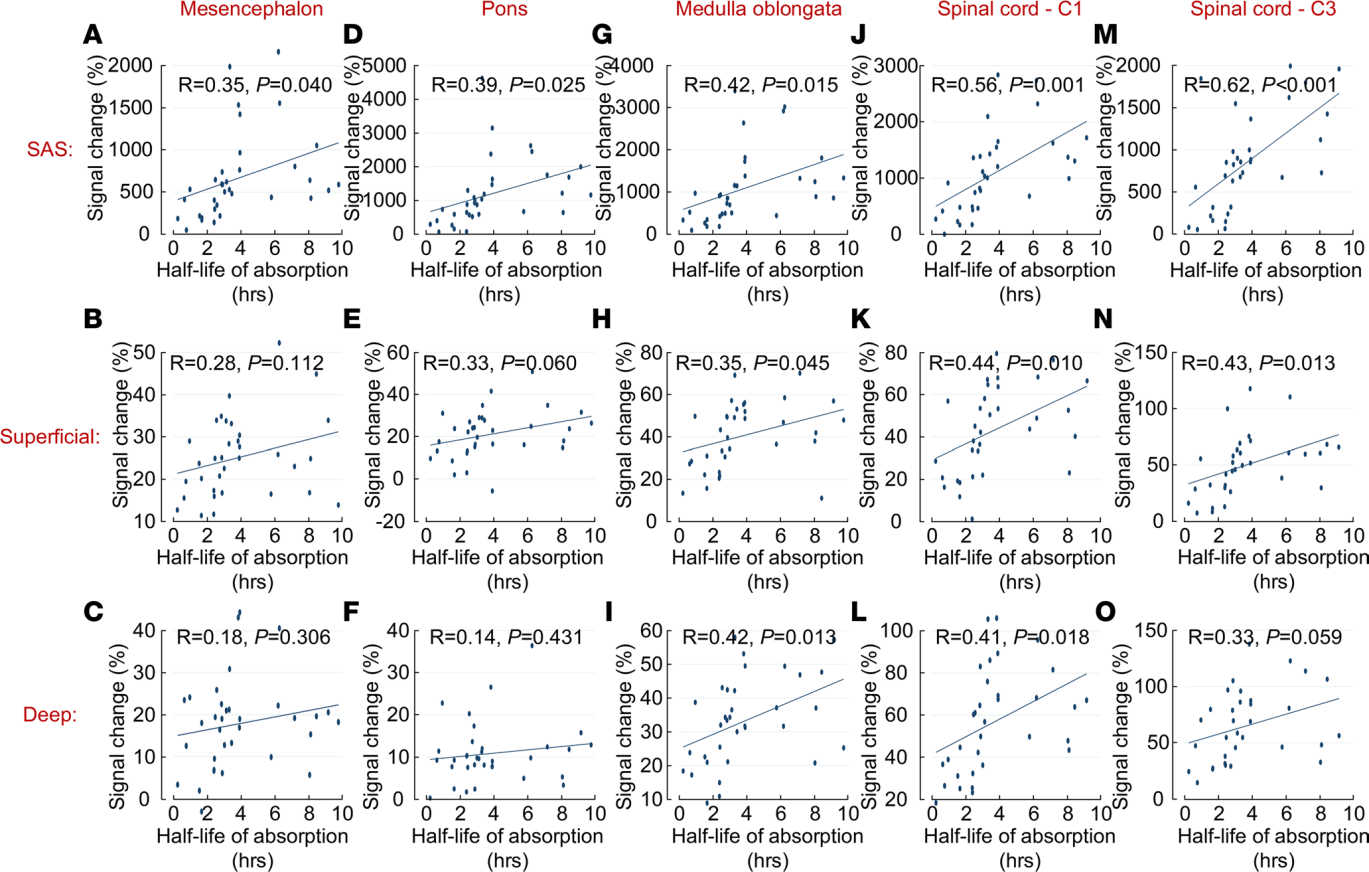
Correlation between CSF-to-blood clearance and tracer enrichment after 24 hours for the subarachnoid space (SAS) and superficial and deep regions of the parenchyma. (**A**–**O**) CSF-to-blood clearance is estimated by half-life absorption. Both reference patients (*n* = 25) and patients with idiopathic normal pressure hydrocephalus (*n* = 8) are included. Pearson correlation coefficients and significance levels are given.

For all patients, there were significant correlations between CSF-to-blood clearance, estimated by T_1/2,__abs_, and enrichment of SAS after 24 hours (Figure 7). The correlation between CSF-to-blood clearance and parenchymal enrichment was stronger in spinal cord and medulla oblongata than in pons and mesencephalon.

## Discussion

The main findings of this study are that a CSF tracer administered at the lumbar level enriched the entire parenchyma of the human upper cervical spinal cord and the brain stem. Tracer enrichment in parenchyma is associated with tracer enrichment in adjacent SAS and the distance from the parenchymal surface. Furthermore, the tracer enrichment in both the parenchyma and the SAS is associated with CSF-to-blood clearance and is altered in iNPH compared with younger reference patients.

### Gadobutrol as a CSF tracer substance.

For this investigation, we used the MRI contrast agent gadobutrol as a CSF tracer substance injected into the SAS at the lumbar level. Gadobutrol is a low-weight (605 Da), hydrophilic molecule that does not pass the BBB. Lipophilic molecules are more rapidly removed from the intrathecal space (18) and do not diffuse as long into the spinal cord as hydrophilic molecules (19). In pigs, 4 different intrathecally administered opioids showed markedly different pharmacodynamic behavior, which in part could be related to differences in hydrophobicity (20). The distribution of tracers in both the brain and the spinal cord in animals is size dependent with slower passage to deeper parenchymal structures for larger molecules compared with smaller molecules (13, 21). The recently discovered subarachnoid lymphatic-like membrane may also restrict the movement of molecules > 3,000 Da within the intracranial SAS (22). Animal studies are essential to understanding physiology, but anatomical differences between species exist. For example, small sensory axons are located in the substantia gelatinosa in the dorsal horn of the spinal cord (23). Sensory impulses are suppressed by morphine injected into this site (24). In rats, this area is 10–20 μm from the spinal cord surface; in dogs, this distance is 200–300 μm; and in humans, it is 500 μm (18). After intrathecal delivery of morphine, the time to onset of effect is 2–3 minutes in mice (25), 30 minutes in cats (26), and more than 1 hour in humans (27). Table 1 summarizes physiochemical and human pharmacokinetic properties of CSF tracers and different intrathecal pharmaceuticals in clinical use. Human in vivo distribution data for the spinal cord for these agents are lacking, but many have approximately the same size as gadobutrol. Depending on other properties, like hydrophobicity, the distribution could be roughly estimated from our data, but intrathecal distribution patterns are complex. To illustrate this, nusinersen, an intrathecally administered antisense oligonucleutide for treatment of SMA, is detectable throughout the CNS in 3 human autopsy cases (15), despite higher molecular weight compared with many other substances (Table 1). Nusinersen is hydrophilic and has a long terminal elimination half-life at 135–177 days (28), which may explain the wide spread of the molecule despite its relatively higher weight.

### Tracer distribution in the SAS.

The fast distribution of the tracer from the lumbar to the cervical level could be explained by a potent dispersion effect in the spinal canal (29). The less tracer enrichment in the interpeduncular fossa and tracer retention at this site after 48 hours is probably a result of increased distance from the injection site (11) and less dispersion of CSF in a more remote space compared with the SAS in the spinal canal (30, 31).

### Tracer distribution in the spinal cord and brainstem.

Our results show that tracer in CSF enriches brainstem and spinal cord centripetally from the periphery to the center. Evidence for this includes: (a) there is a faster and stronger enrichment in the superficial regions compared with the deep regions (Figure 3); (b) for the largest structure, pons, the correlation between the tracer enrichment in the SAS and the superficial regions is slightly stronger than the correlation between the SAS and the deeper regions (Figure 4); and (c) the differences in parenchymal enrichment between superficial regions and deep regions are more prominent in larger structures — e.g., pons, compared with smaller structures, such as the spinal cord (Figures 3 and 4).

Based on previous animal studies, the tracer probably moves through perivascular spaces in the parenchyma in a centripetal direction, not through the central canal from the fourth ventricle (11, 13), but how the tracer moves from the SAS to the deep regions of the spinal cord is not possible to assess with the spatial resolution in this study (1 mm voxel size). Apart from perivascular bulk flow, diffusion through the parenchyma is probably also involved (9).

### Clearance of tracer.

Clearance (T_1/2,__abs_) to the blood and the tracer enrichment in the SAS and in the spinal cord are highly associated (Table 3 and Figure 7); the pharmacokinetic model for CSF to blood clearance provides an estimate of how fast a substance is cleared to the blood, which in turn can estimate the remaining tracer levels in the SAS and parenchyma. We injected the tracer into the SAS and followed the tracer from the SAS into the parenchyma and to the blood. Whether the tracer, when in the spinal cord and brain stem, then returns to SAS through the parenchyma along perivascular spaces and to which extent it is cleared through other routes — e.g., directly to the blood — is not possible to assess with our methodology. The blood-spinal cord barrier is more permeable to tracers compared with the BBB (32) and a tracer injected into the SAS may pass to the vessel lumen (13). As proposed in refs. 13 and 31, substances can possibly pass over the vessel wall from CNS to blood even if passage the other way is restricted. Most gadobutrol injected into the SAS at the lumbar level exits in the SAS before it arrives outside the upper brain convexities, indicating that gadobutrol is mostly cleared at the spinal level, with maximum tracer levels in blood occurring much earlier than in the parasagittal dura mater (30, 33). In mice, tracers in the CSF pass to spinal lymphatic vessels outside of the dura mater (34). In humans, molecules in CSF may pass to lymphatic vessels in the dura mater at the convexity of the skull (35), but other CSF outflow routes also may exist, and a quantitative comparison of different CSF outflow routes in humans is lacking (36). CSF efflux to meninges and bone marrow enables for CNS-immune crosstalk (37–39). Our current findings suggest immunologically derived solutes, and perhaps cells, may also access the spinal cord and brain stem.

### Tracer distribution in patients with iNPH.

The delayed enrichment of tracer in the parenchyma and slower CSF clearance in the iNPH group compared with the reference group could be related to both disease and age. In patients with iNPH, the clearance from the brain was reduced (8), but patients in the iNPH group in our study were significantly older, and age is correlated to higher CSF tracer levels and decreased CSF clearance (33). The delayed clearance from the parenchyma in the iNPH group could be explained by reduced clearance from the SAS. With more tracer available in the SAS for a longer period, it is reasonable to expect more tracer in the nearby parenchyma. Clearance from the parenchyma to the SAS is not possible to address with the temporal resolution of this study. To assess the clearance from the parenchyma, injection of tracer directly into the spinal cord parenchyma would be preferred, but that is not feasible in humans in vivo. To this end, since CSF clearance differs between patient groups and at an individual level (40), dosage of intrathecal drugs should be individually tailored by assessing CSF clearance capacity before treatment (33).

### Limitations.

Patients with tentative CSF disorders were investigated in this study with off-label intrathecal use of gadobutrol. Patients without any diagnosis of CSF disorder after assessment at the neurosurgery department were assigned reference patients. While a healthy age-matched control did not exist, we consistently observed tracer enrichment in the spinal cord and brain stem for both groups. For practical reasons, the upper cervical spine and not the entire spinal cord was investigated. The abrupt signal change at the interface between the SAS and the parenchyma may result in Gibbs artifacts in the spinal cord and biased measurements (41). To minimize this, obvious artifacts and measurements in the most superficial parts of the parenchyma were avoided. Also, when artifacts were suspected, the regions of interest (ROIs) were placed in the inflection point of the wave-formed artifacts for the most accurate measurements (41).

Effects of sleep and arousal state for clearance from the parenchyma and CSF were not assessed in this study. In a previous study by our group, with the same method as in the present study, we showed delayed clearance of tracer from the brain, but not from the CSF, after 1 night of total sleep deprivation (42). In a more recent modeling study, which also added the volumes of different brain regions, there was no significant difference between the 2 groups after 24 hours, but groups were significantly different after 48 hours (43). Animal studies have so far shown more prominent effects of sleep for clearance from the brain and CSF (44, 45). All patients stayed at the hospital for 2 nights in a similar setting, and we see it very unlikely that any minor differences in sleep between the groups could have an effect on the present results.

### Implications.

Altogether, the main implication of our study concerns the potential of distributing drugs in CSF to bypass the barrier constituted by the blood vessel walls and reach the extravascular compartment via CSF from the spinal cord and brainstem surface. The pharmacokinetics of gadobutrol could be used as a rough estimate of the pharmacokinetics for an agent with similar properties. In elderly patients with iNPH, delayed clearance from the SAS and higher amounts of tracer were detected in the parenchyma, which should be considered in intrathecal therapy regimens. The wide parenchymal distribution of this CSF tracer could also apply to antigens and possible immune cells, suggesting CSF as an important mediator between the spinal cord and the immune system in the meninges and the bone marrow.

### Further work.

In animal studies, the delivery of intrathecal drugs to the CNS via the glymphatic system can be enhanced by systemic drugs (46, 47) and ultrasound (48). The physiochemical complexity, the possibility to modulate the distribution of agents in the thecal space, and differences between species emphasize the importance of further human CSF tracer studies. This could include studies with tracers of varied sizes, addition of systemic drug therapy, and imaging of the entire spinal cord. MR sequences able to quantify the amount of gadolinium are evolving (49) and could be useful for pharmacokinetic assessment of tracer concentrations in the spinal cord.

### Conclusion.

Intrathecally administered gadobutrol, a small molecular weight CSF tracer, was shown to enrich all ROIs in the upper spinal cord and brain stem from the surface. Drugs with features like gadobutrol can, thus, also be expected to access the extravascular compartment of the spinal cord and brain stem from CSF; however, with different clearance profiles between patients and patient groups. To this end, CSF clearance capacity can be measured by assessing tracer pharmacokinetics from CSF to blood, which may guide dosage of intrathecal treatment regimes.

## Methods

### Patients

This prospective, observational study includes patients examined with MRI after intrathecal contrast injection for evaluation of a tentative CSF disorder. All patients were referred to the neurosurgery department at Oslo University Hospital-Rikshopitalet and were included consecutively before the MRI examinations and the clinical evaluation. Exclusion criteria included patient ages < 18 years or > 80 years, pregnancy, breastfeeding, a previous adverse reaction to a contrast agent, a history of severe allergic reactions, or renal dysfunction.

### MRI protocol

All image data were obtained with the same 3 Tesla Philips Ingenia MRI scanner (Philips Medical Systems) with equal imaging protocol settings at all time points: 3D T1-weighted image (T1 magnetization-prepared rapid gradient-echo [MPRAGE]) in sagittal plane; time to inversion: 853 ms; repetition time: “shortest” (typically 5.1 ms); echo time: “shortest” (typically 2.3 ms); flip angle: 8 degrees; field of view: 256 × 256 cm; and matrix: 256 × 256 pixels (reconstructed to 512 × 512 pixels). We sampled 184 overlapping 1 mm isotropic voxels, which were automatically reconstructed to 368 slices with 0.5 mm thickness. Acquisition time was 6 minutes and 29 seconds.

### Intrathecal administration of tracer

Approximately at 9 a.m., after the first MRI scan (baseline), 0.5 mmol gadobutrol (Gadovist, Bayer) was injected after correct needle position in the SAS was confirmed by CSF backflow or injection of 3 mL iodine contrast agent. Before leaving the intervention suite, the patient rotated along the long axis of the body and was instructed to remain in a supine position until approximately 4 p.m. the same day.

### MRI acquisitions

Each patient was examined with the same image parameters before contrast, 10–15 minutes after contrast, 2–4 hours after contrast, 4–7 hours after contrast, and approximately 24 and 48 hours after contrast. For practical reasons, the time after contrast for each patient varied to a moderate degree.

### Image analysis

All image data were obtained from the hospital’s picture and archiving system (PACS), Sectra, version 7. No preprocessing of the images was performed. Fifteen ROIs were placed in the following locations (Figure 1). ROIs in the mesencephalon include those (a) in the center between the aqueduct and the interpeduncular cistern; (b) in the anterior part of the right cerebral peduncle; and (c) in the interpeduncular cistern. ROIs in the pons include those (d) to the right in the anterior part; (e) in the center; and (f) in the right lateral cerebellomedullary cistern. ROIs in the medulla oblongata include those (g) in the center; (h) in the right anterolateral part; and (i) in the adjacent SAS. ROIs in the spinal cord, C1 level, include those (j) in the center; (k) in the right anterolateral part; and (l) in the adjacent SAS. ROIs in the spinal cord, C3 level, include those (m) in the center; (n) in the right anterolateral part; and (o) in the adjacent subarachnoid space.

The signal from the spine decreases caudally with increased distance from the head coil. The C3 level was chosen as the most caudal level to get an acceptable signal/noise ratio in all patients. To correct for image grayscale changes after the contrast administration, we normalized the signal intensity for each image of the same patient by use of signal intensity of retrobulbar fat before and after contrast as reference tissue. Retrobulbar fat is not expected to enhance after intrathecal contrast injection and, therefore, should serve as a consistent reference tissue throughout all time points.

Although the signal increase from the tracer in both the SAS and the parenchyma is easily recognized on the MRI images, the data are not transferable to quantifiable concentrations of the tracer. Instead, we used the signal increase in the images as a semiquantitative measurement of tracer distribution.

### Tracer analysis in blood

Pharmacokinetic model-estimated CSF-to-blood clearance variables were available for 11 patients with iNPH and 26 reference patients; the method has previously been described in detail (33, 40). In short, venous blood samples for each patient were collected in conjunction with the MRI examinations and kept in a freezer. After preparation of the samples and quality control of the method, the concentrations of gadolinium in the samples were quantified. A pharmacokinetic model was developed to calculate the parameters in Table 3.

### Statistics

Statistical significance was accepted at the 0.05 level (2 tailed). Continuous data presented as mean ± SD, differences of iNPH from the REF group were determined by 2-tailed independent-sample *t* tests for continuous variables, by Fisher’s exact test for categorical variables, and correlation between continuous variables was assessed by Pearson’s corSperelation coefficient. We assessed the repeated measurements of percentage change in normalized T1-signal over time with linear mixed models by maximum likelihood estimation using a subject-specific random intercept and distinct residuals before and after 48-hour follow-up. Using estimated marginal means from the statistical model, we tested the difference between groups at each time point and presented results with mean and 95% CI.

### Study approval

The study was approved by the following institutions: The Regional Committee for Medical and Health Research Ethics of Health, Southeast Region, Norway (2015/96); the Institutional Review Board of Oslo University Hospital (2015/1868); and the National Medicines Agency, Norway (15/04932-7). Written and oral informed consent were provided by all patients before inclusion.

### Data availability

The source data for Figures 2–7 are presented in the Supporting Data Values File (supplemental material available online with this article; https://doi.org/10.1172/jci.insight.173276DS1). Other data analyzed in this study are available from the corresponding author on reasonable request.

## Author contributions

EM, PKE, and GR conceptualized and designed the study. EM, PKE, and GR provided investigation and formal analysis. PKE and GR supervised and administered the study. EM, PKE, and GR wrote the original draft. AHP performed statistical analysis. EM, AHP, PKE, and GR wrote, reviewed, and edited the manuscript. All authors (EM, AHP, PKE, and GR) approved the final manuscript.

## Supplementary Material

ICMJE disclosure forms

Supporting data values

## Figures and Tables

**Table 3:**
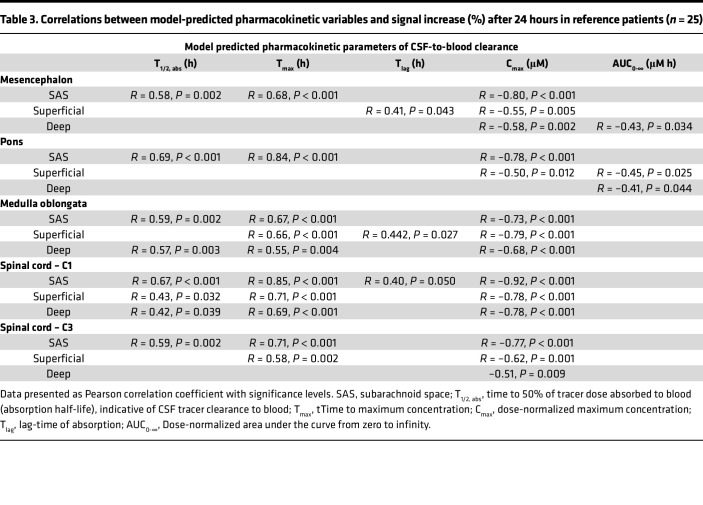
Correlations between model-predicted pharmacokinetic variables and signal increase (%) after 24 hours in reference patients (*n* = 25)

**Table 2:**
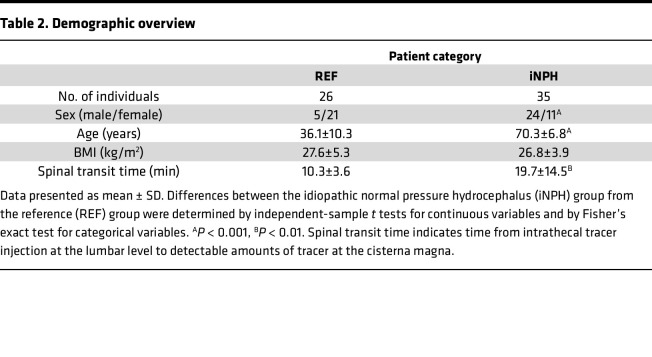
Demographic overview

**Table 1:**
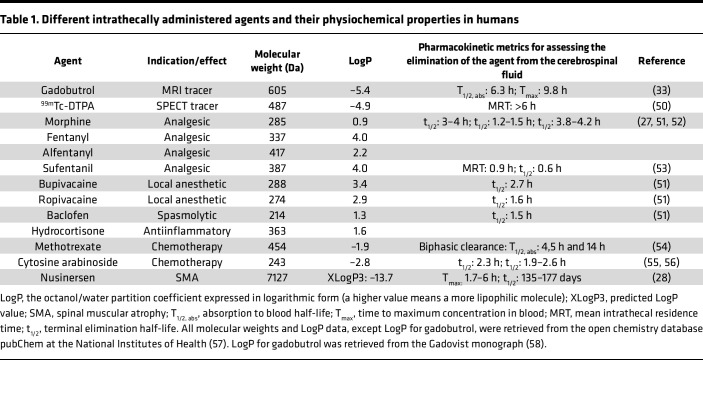
Different intrathecally administered agents and their physiochemical properties in humans

## Version 1 12/08/2023

Electronic publication
